# Drug Repurposing Approaches to Combating Viral Infections

**DOI:** 10.3390/jcm9113777

**Published:** 2020-11-23

**Authors:** Jay Trivedi, Mahesh Mohan, Siddappa N. Byrareddy

**Affiliations:** 1 Department of Pharmacology and Experimental Neuroscience, University of Nebraska Medical Centre, Omaha, NE 68198, USA; 30.jaytrivedi@gmail.com; 2 Texas Biomedical Research Institute, Southwest National Primate Research Center, San Antonio, TX 78227, USA; 3 Department of Genetics, Cell Biology and Anatomy, University of Nebraska Medical Centre, Omaha, NE 68198, USA; 4 Department of Biochemistry and Molecular Biology, University of Nebraska Medical Centre, Omaha, NE 68198, USA

**Keywords:** COVID-19, drug repurposing/reprofiling, HIV, USFDA, zidovudine

## Abstract

Development of novel antiviral molecules from the beginning costs an average of $350 million to $2 billion per drug, and the journey from the laboratory to the clinic takes about 10–15 years. Utilization of drug repurposing approaches has generated substantial interest in order to overcome these drawbacks. A drastic reduction in the failure rate, which otherwise is ~92%, is achieved with the drug repurposing approach. The recent exploration of the drug repurposing approach to combat the COVID-19 pandemic has further validated the fact that it is more beneficial to reinvestigate the in-practice drugs for a new application instead of designing novel drugs. The first successful example of drug repurposing is zidovudine (AZT), which was developed as an anti-cancer agent in the 1960s and was later approved by the US FDA as an anti-HIV therapeutic drug in the late 1980s after fast track clinical trials. Since that time, the drug repurposing approach has been successfully utilized to develop effective therapeutic strategies against a plethora of diseases. Hence, an extensive application of the drug repurposing approach will not only help to fight the current pandemics more efficiently but also predict and prepare for newly emerging viral infections. In this review, we discuss in detail the drug repurposing approach and its advancements related to viral infections such as Human Immunodeficiency Virus (HIV) and Severe Acute Respiratory Syndrome Coronavirus 2 (SARS-CoV-2).

## 1. Introduction

**Figure 1:**
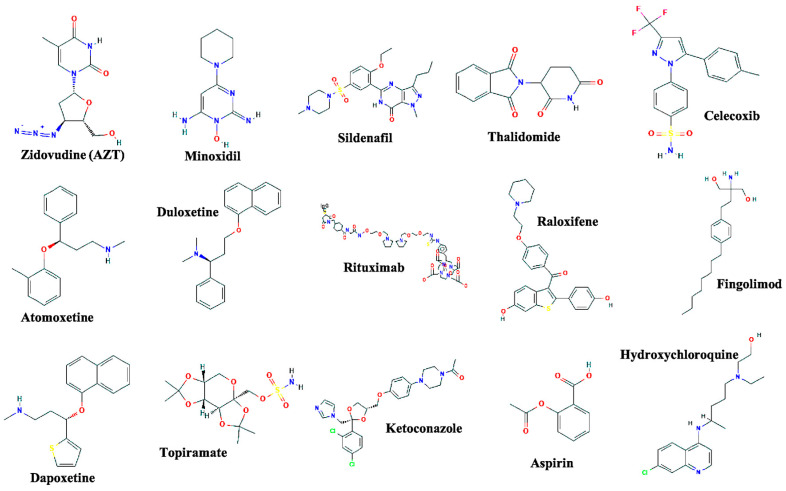
Chemical structures of the drugs that are efficacious examples of drug repurposing approach and are approved by the FDA for their multiple applications. The chemical structures are adapted from (pubchem.ncbi.nlm.nih.gov/).

The drug repurposing approach, also referred to as drug reprofiling or drug repositioning, utilizes mechanistic details of the existing drugs to investigate their novel applications against other disease conditions. Given the high failure rate of ~90% and the enormous amount of resources associated with the development of novel drugs and their combinations, the drug repurposing approach has gained a substantial amount of attention among the scientific community. With this approach, the risk of failure is drastically reduced since the drugs being investigated have already been deemed to be safe for translational applications in humans and other diseases; therefore, the toxicity factor associated with these drugs can be eliminated to a great extent. Second, this approach may also drastically reduce the time required for preclinical evaluation, clinical trials, as well as the development of various drug formulations based on structure-activity relationship (SAR) [1,2]. Further, the possible reduction in the duration of testing may potentially enable us to rapidly provide potent drug formulations to the required communities and pandemic areas around the world. Although this approach is far from a newly developed method, it has obtained considerable momentum, especially during the development of treatment strategies to combat the morbidity and mortality caused by viruses, such as the Human Immunodeficiency Virus (HIV) and Severe Acute Respiratory Syndrome Coronavirus 2 (SARS-CoV-2). The approach has been utilized in the development of more efficient therapies and has permitted the development of several drugs for their new applications (Figure 1).

Apart from the applications as antiviral molecules, the drug repurposing approach is being actively investigated for development of drugs for other emerging infectious diseases [3,4,5]. The clinical evaluation of the approved drugs for their new application requires fundamental understanding of pharmacokinetics and pharmacodynamics. The large amount of existing Phase IV and post-marketing data offers an extensive understanding in terms of drug candidate selection for the new application [6]. Giovannoni et al., reviewed the policy-oriented approaches for drug repurposing and highlighted the vital importance of post-marketing studies for early and fast track approval of the drugs during pandemics [7]. The pharmaceutical companies often focus on the post-marketing follow-up studies as a part of life cycle management (LCM) through which the companies often attempt to extend the patent life of their drugs. However, these studies become enormously useful for the repurposing of the existing drugs in the long term. A timeline of the drugs that are approved by US FDA for the novel indications such as cancer, obesity and autoimmune diseases are highlighted in Table 1.

**Figure 2:**
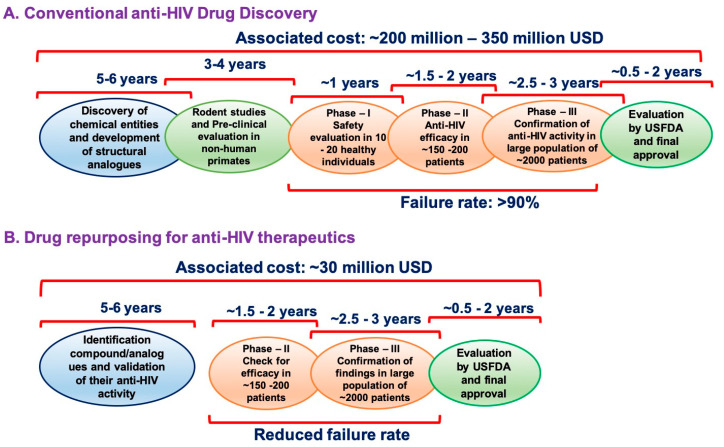
Schematic representation of time and resources associated with conventional development and characterization of novel anti-HIV drugs (**A**) versus drug repurposing approach (**B**).

Despite enormous advances in pharmaceutical technologies and our understanding of disease transmission and pathogenesis, the failure rates of drugs have amplified in the past few decades [23,24]. The primary factors in the decline of success rate are time, cost, and resources associated with the journey of a drug from the laboratory to the clinic. Multiple statistical studies on global research and development (R&D) expenditure has estimated an approximate cost of $350 million to $2 billion per drug for development and its clinical trials; a grueling process that takes approximately 15–17 years [25] [Figure 2]. Although the total global R&D expenditure has increased almost tenfold from $4 billion in 1975 to $41 billion in 2010, the approval rate of new drugs by the FDA has remained more or less stagnant [26,27,28]. In total, 26 new drugs were approved by the FDA in 1975, whereas, 27 new drugs were approved in 2013, which further dropped to 22 new approvals in 2016. The decline in the approval rates was largely due to the increased failure of drugs at various stages of their clinical trials [29]. Hence, repurposing or reprofiling of drugs has gained attention in recent times for both commonly occurring as well as rare infectious diseases.

Several commercially available chemical libraries contain established drugs, that are of enormous advantage in disease biology and computational biology. These chemical libraries have accelerated the progressive applications of the drug repositioning approach. The activity-based approach, which primarily involves screening of large-scale libraries, is laborious, though it is more advantageous and reliable compared to in silico computational analysis due to fewer false positives. It might also provide information about the effects associated with possible primary or secondary drug metabolites, which is not possible to predict with the computational approach (Table 2). On the other hand, the in silico computational approach helps us to quickly obtain potential targets for further investigations.

Activity-based retasking of drugs provide several advantages over designing novel molecules including lower risk of failure, rapid availability of drugs to the community, as well as a drastic reduction in overall cost associated with drug development.

## 2. The Origin of Drug Repurposing Approach: Zidovudine as an Example

The first FDA approved anti-HIV medication, zidovudine, was initially developed as an anti-cancer medicine in the late 1960s before being developed as an anti-HIV formulation as a result of a large-scale library screening approach to fast-track preclinical trials [8]. Tamin and Baltimore revolutionized the field of virology and molecular biology by their discovery of reverse transcription (RT) and DNA provirus in the 1960s and strengthened the theory of that time that most cancers are caused by environmental viruses. Extending to these groundbreaking discoveries, several scientists demonstrated that the molecules that were blocking the nucleotide synthesis were not only proven to be used as anti-cancer drugs but also potent antibacterial as well as antiviral drugs. These discoveries eventually led to the development of zidovudine (AZT) [Figure 1] by Horwitz in 1964 as a potent anticancer agent [30]. A decade later, Ostertag et al., demonstrated that zidovudine specifically inhibited the murine leukemia virus (MLV) by blocking the virus life cycle at a very early stage [31]. This discovery gained scientific attention immediately as MLV is a retrovirus; a few years down the line, the identification of HIV, another retrovirus as a causative agent of AIDS was demanding the immediate therapeutic development to control the pandemic created by HIV. In late 1985, AZT was reported to block HIV replication in vitro [32], and soon after this study was published AZT received the approval from FDA for large scale clinical trials. Oral administration of AZT in 282 patients showed a reduction in viral load and stability in the CD4+ T-cell count [8]. This was not only the first successful example of drug repurposing, but also the shortest duration of drug approval from its first report, which was completed in 25 months. These studies indicated that the drug repurposing approach may be extremely vital to save time as well as resources associated with developing new drugs. Recent documents on the development of multidrug-resistant HIV strains, even in anti-retroviral naïve patients, have raised alarming signals in the scientific community and promoted the rapid need to update the current antiretroviral therapeutic strategies [33]. Application of drug repurposing approaches to develop anti-HIV molecules would inspire not only the development of more efficient anti-HIV formulations, but also make them rapidly available to vulnerable populations, especially those in remote areas of the developing world.

In the case of HIV and other retroviruses, extremely fast rate of replication coupled with the lack of 3′–5′ exonuclease proofreading activity allow them to develop drug resistant mutations at a very rapid rate. Hence, targeting the viral enzymes allowed the virus to overcome the inhibitory potential of drugs and develop resistance due to the hypermutations generated during reverse transcription. Additionally, with limited resources of its own, HIV as well as other viruses are inefficient at replicating within the host system. Therefore, viruses hijack the host machinery not only to successfully propagate in host cells but also evade the host immune system. Various studies have demonstrated the vital role of these host factors in the virus life cycle and have highlighted them as potential therapeutic targets. Hence, targeting cellular HIV dependency factors (HDFs) along with the viral enzymes to attenuate HIV replication may help overcome this drawback of the emergence of drug resistance and strengthen our efforts to eliminate the virus [34,35].

During the past two decades, a sizeable number of host factors have emerged as potential cellular targets for anti-HIV therapeutics [29,35,36,37,38,39,40,41,42,43,44,45,46] and, of particular interest, for drug repurposing approaches. Drug repurposing incorporates various approaches including large-scale screening of chemical libraries as well as in silico computational approaches. Development of primary active scaffolds will lead to structural formulations that are more specific and less toxic. Drug repurposing approaches to identify and characterize the HDF(s) as potential therapeutic targets would be more advantageous compared to targeting the viral proteins to overcome the drawback of current therapeutics including drug resistance and toxicity to the host.

## 3. Anti-HIV Therapeutics as Drug Repurposing Candidates

The bottleneck steps in the virus life cycle such as reverse transcription, integration, and maturation are conserved across the *Retroviridae* family to a substantial extent. Hence, molecules that inhibit HIV-1 replication would likely inhibit other retroviruses and vice versa. Since the approval of the first anti-HIV medicine, the development of novel anti-HIV therapeutics has erupted including novel chemical entities and structures with significant anti-HIV activities. Not only are these novel formulations used for their anti-HIV activity, but also studied for their different therapeutic implications including various types of cancers. In the late 1990s, the HIV-1 reverse transcriptase inhibitors (RTIs) were highlighted in several reports and demonstrated very efficient anti-HIV activity. Along with the drastic decline in the free circulating viral load, patients treated with RTIs were also observed to have a gradual increase in their CD4^+^ T-cell counts and, more importantly, the treatment also helped control Kaposi’s Sarcoma (KS) [47]. These observations gave the first indications of possible anti-cancer activity of RTIs. RTIs are nucleotide analogs, which hamper cellular DNA synthesis thus inducing cell death even in uninfected cells at high concentrations. For example, cidofovir and ganciclovir have been widely investigated for their ability to induce cell death in rapidly dividing cancer cells [48,49,50]. Efavirenz has been demonstrated to have profound antiproliferative activity against pancreatic cancer as well as anaplastic thyroid cancer [51]. Rilpivirine was recently shown to block Zika virus infection in the brain along with etravirine and efavirenz [52]. However, other FDA approved drugs and investigational RT inhibitors have not been well studied for their anti-cancer activity. Hence, we believe that several of these HIV-1 RT inhibitors should be investigated further for their anti-cancer activities, which may aid in the development of more efficient therapeutic strategies against various incurable cancers. 

Further, the introduction of HIV-1 protease (PR) inhibitor, saquinavir, in December 1995, opened a horizon of opportunities for studying its application not only as an anti-HIV agent, but also anti-cancer and anti- inflammatory agent. Apart from their anti-cancer activity, HIV-1 protease inhibitors such as ritonavir and lopinavir are documented to have anti-protozoal activity at micromolar to nanomolar concentrations [53]. Further, these inhibitors have been reported to have anti-malarial activity [54] and also shown to cure Chagas’ diseases [55]. However, these protease inhibitors demonstrated off-target effects, which have consequently limited their use as medications at various phases of clinical trials for several other diseases.

Most recently, the use of anti-HIV PR inhibitors has shown new hope in the development of effective treatment against the novel SARS-CoV-2 infection (COVID-19). Previously, ribavirin, lopinavir in combination with ritonavir demonstrated significant anti-viral potential against SARS as well as MERS associated coronaviruses [56]. As an extension of these observations, remdesivir was recently tested for its antiviral activity against SARS-CoV-2 in vitro and was found to block virus replication at nanomolar concentration [57]. Additionally, remdesivir also demonstrated strong inhibitory potential against a range of viruses including Filoviruses such as Ebola [58,59,60]. These studies collectively suggest that the FDA approved anti-HIV therapeutic candidates should be considered more actively for additional applications not only for other viruses but also non-infectious diseases such as cancer.

## 4. Drug Repurposing Approach at Present: Against COVID-19

Most recently, lopinavir, remdesivir and hydroxychloroquine (HCQ) were suggested to have significant in vitro inhibitory potential against SARS-CoV-2, the causative agent of COVID-19 that has caused major devastation across the globe [57,61,62,63]. Repurposing of anti-hepatitis C virus (HCV) molecules as anti-SARS-CoV-2 is also being actively considered to control disease progression [64]. Additionally, the protease inhibitors [65], anti-inflammatory drugs [66], and anti-aging drugs [67] are actively being considered for the development of potential therapeutic strategies against COVID-19 to overcome the pandemic. A summary of the drugs that are at various stages of their clinical trials for COVID-19 is summarized in Table 3.

Previously, chloroquine (CQ) derivatives had been tested on coronaviruses and demonstrated a potential antiviral effect in-vitro [68], which were further supported by the recent findings where HCQ was demonstrated to inhibit COVID-19 replication in vitro [61,62]. Initially, HCQ demonstrated promising antiviral effects in patients suffering from severe acute pneumonia infected with SARS-CoV-2 [69,70,71], which led to the fast-track approval of HCQ for COVID-19 patients by the USFDA. However, subsequent reports of moderate or no effect of HCQ questioned its use in COVID-19 patients and eventually its retraction after two pioneering studies demonstrated reduced antiviral but more adverse effects of HCQ in patients raising alarming signals over the effectiveness of HCQ against COVID-19. Further, the use of intravenous immunoglobulins for their ability to produce anti-inflammatory and immunomodulatory effects is being evaluated in the patients suffering from pneumonia caused by COIVD-19 (NCT04303507). Subsequently, HIV-1 protease (PR) inhibitors have gained a popularity in the scientific community for their inhibitory potential against COVID-19. Several of the HIV-1 reverse transcriptase (RT) inhibitors including remdesivir (NCT04257656, NCT04252664), umifenovir (NCT04260594) are being evaluated either individually or in different combinations in the patients suffering from COVID-19. Additionally, other gold-standard medications such as Thalidomide (NCT04273581, NCT04273529) as well as vitamin-C (NCT04264533) are being investigated actively in patients suffering from severe COVID-19 disease. Furthermore, other pharmacologically active molecules such as methylprednisolone (NCT04244591), pirfenidone (NCT04282902), bromhexine hydrochloride (NCT04273763), bevacizumab (NCT04275414), fingolimod (NCT04280588) have occupied a prominent place as potential therapeutic candidates in patients suffering from critical illness due to the COVID-19 associated complications (Table 3). These studies collectively validated the fact that the utilization of the drug repurposing approach will not only monumentally reduce the recourse consumption, but also help to drastically reduce the failure rate.

Along with these clinical trials, several other studies documented the critical host factors and their modulation by small molecules as a potential therapeutic approach. For instance, Clausen et al., demonstrated that the entry of SARS-CoV-2 into the host cells was dependent on heparan sulfate present on the cell surface [72]. Further, Zhang et al., performed drug repurposing screening targeting heparan sulfate mediated endocytosis and identified several candidate drugs including, mitoxantrone, sunitinib and BNTX that inhibited SARS-CoV-2 replication in vitro at concentrations as low as 10 nM [73]. Lactoferrin was shown to inhibit SARS-CoV expression both in vitro as well as in vivo and is being further investigated for its antiviral efficacy against SARS-CoV-2 [74]. Quercetin, an established flavonoid, demonstrated significant synergistic antiviral activity in association with vitamin C (ascorbic acid) supporting further therapeutic use of this combination as a potential therapeutic against SARS-CoV-2 [75].

## 5. Drawbacks of Drug Repurposing

Since the new applications are found based on the previously known data such as pharmacokinetics and mechanism of action, the time for clinical evaluations for a new application of a drug is reduced. However, certain drawbacks need to be considered during the utilization of this approach. The primary concerns associated with repurposing existing drugs is the intellectual property rights and additional national, as well as international legislations associated with the drug patents. These are the major obstacles of investigating the novel applications of previously known drugs. Further, if the drugs or their previous applications are patented, then that provides the original developers the market exclusivity. This not only makes the data unavailable, but also makes the molecule or chemical scaffolds unavailable for further investigation. An extension of this, deviating from the chemical structure based on structure-activity relationship violates the principle of drug repurposing and makes the new chemical entity subject to the dogma of pre-clinical and clinical evaluation. These drawbacks associated with the drug repurposing should be addressed scientifically as well as legally to utilize the approach to its maximum potential and make the drugs rapidly available during global pandemics and worldwide emergencies. Furthermore, the previous application of drug/s are reported at a particular dose range, which may not necessarily be effective for its new application. Hence, it is critical to determine the effective dose range of a drug/s for its new application/s along with the determination of toxicity and off-target effects at the effective dose. Despite the associated advantages and promising results over the period, global financial support for the drug repurposing approach has been lacking. Finally, the low market price of the drugs, shorter duration of the patent with new applications, and low returns on the investments are the primary reasons why pharmaceutical companies are not extensively interested in the drug repurposing approach. 

## 6. Conclusions

With the current arsenal of antiviral therapeutic regimens, we have successfully controlled the major pandemics including AIDS. However, the deaths associated with viral infections such as HIV and COVID-19 remain major concerns. Repurposing of the drugs has always been of major interest due to the advantages associated with the approach including reduced failure rates and decreased time as well as resource consumptions. The first successful approval of drug repurposing was also the first approved anti-HIV medicine, which was initially developed as an anti-cancer drug in the 1960s. The recent exploitation of the drug repurposing approach to find efficient therapeutics against COVID-19 has highlighted the fact that designing novel molecules during global pandemics is less advantageous over retasking of drugs.

Designing novel antiviral molecules aimed to target host or viral factors is expensive and takes nearly a decade from design to therapeutic application. Therefore, drug repurposing has gained popularity not only in the scientific community, but also in the pharmaceutical industries. Activity-based drug repurposing is more beneficial compared to the *in-silico* approach as it has a lower chance of obtaining the targets as false hits. Hence, the drug repurposing approach should be reconsidered for fast-track development of better antiviral therapeutic strategies to combat viral pandemics such as AIDS, as well as COVID-19 and other diseases. Additionally, while exploring the drug repurposing approach, other challenges associated with intellectual property rights should also be considered before beginning future studies.

## Figures and Tables

**Table 1:** Overview of selected successful drug reprofiling candidates.

Drug Name	Original Indication	New Indication	Year of Approval
Zidovudine	Cancer	AIDS	1987 [8]
Minoxidil	Hypertension	Hair loss	1988 [9]
Sildenafil	Angina	Erectile dysfunction	1998 [10]
Thalidomide	Morning sickness	Erythema nodosum leprosum and multiple myeloma	1998 [11] and 2006 [12]
Celecoxib	Pain and inflammation	Familial adenomatous polyps	2000 [13]
Atomoxetine	Parkinson disease	ADHD	2002 [14]
Duloxetine	Depression	SUI	2004 [15]
Rituximab	Various cancers	Rheumatoid arthritis	2006 [16]
Raloxifene	Osteoporosis	Breast cancer	2007 [17]
Fingolimod	Transplant rejection	MS	2010 [18]
Dapoxetine	Analgesia and depression	Premature ejaculation	2012 [19]
Topiramate	Epilepsy	Obesity	2012 [20]
Ketoconazole	Fungal infections	Cushing syndrome	2014 [21]
Aspirin	Analgesia	Colorectal cancer	2015 [22]

AIDS = Acquired Immunodeficiency Syndrome. ADHD = Attention deficit hyperactivity disorder. SUI = Stress urinary incontinence. MS = Multiple Sclerosis.

**Table 2:** Pros and cons associated with activity-based and in silico drug repositioning approaches.

Approach	Advantages	Disadvantages
Activity-based approach	No limitation for in vitro cell-based as well as cell-free target-based screening assays	Time and labor-consuming and required highly skilled individuals
	Easy to validate screening hits	Requires a large collection of existing drugs
	Reduced chances of false-positive hits during the screening	Requires the development and optimization of efficient screening assays
	Molecules with activities due to primary and secondary metabolites are also obtained	
In silico approach	Not time and labor efficient	Requires detailed structural insight of target proteins both in normal as well as diseased conditions
	No need for an entire collection of existing drugs	Increased rates of false-positive hits during the screening
		No need to develop a screening assay

**Table 3:** Summary of drugs being repurposed in clinical trials against COVID-19.

Therapeutic Intervention	Class of the Drug/s	Clinical Condition/s of the Participants of the Trial	Trial Identification Number *	Phase
Hydroxychloroquine	Antimalarial and amebicide	30 patients suffering from pneumonia due to COVID-19	NCT04261517	3
Chloroquine	Antimalarial and amebicide	10,000 patients in a prophylaxis study for COVID-19	NCT04303507	N/A
Human immunoglobulin	Antibody	80 patients suffering from pneumonia due to COVID-19	NCT04261426	2 and 3
Remdesivir	Nucleotide reverse transcriptase inhibitor	452 patients suffering from a severe respiratory infection due to COVID-19	NCT04257656	3
Remdesivir	Nucleotide reverse transcriptase inhibitor	308 patients with mild or moderate respiratory tract infection caused by COVID-19	NCT04252664	3
Arbidol (umifenovir)	Virus entry (Fusion) inhibitor	380 patients suffering from Pneumonia caused by COVID-19	NCT04260594	4
Arbidol or lopinavir-ritonavir or oseltamivir	Combination of virus entry (Fusion) inhibitor and protease inhibitor	400 patients infected with COVID-19	NCT04255017	4
Arbidol or lopinavir + ritonavir	Combination of virus entry (Fusion) inhibitor and protease inhibitor	125 patients infected with COVID-19	NCT04252885	4
Darunavir + cobicistat	Protease inhibitor (Darunavir) in combination with Booster (cobicistat, a CYP3A inhibitor)	30 patients suffering from Pneumonia caused by COVID-19	NCT04252274	3
TCM combination with lopinavir + ritonavir, α-interferon via aerosol	Cytokine in combination with protease inhibitor	150 patients infected with COVID-19	NCT04251871	N/A
Recombinant human interferon α2β	Cytokine	328 patients infected with COVID-19	NCT04293887	1
Carrimycin or lopinavir + ritonavir or arbidol or chloroquine phosphate	Antibiotic in combination with booster (arbidol) or antimalarial/ amebicide	520 patients infected with COVID-19	NCT04286503	4
Danoprevir + ritonavir + interferon inhalation or lopinavir + ritonavir or TCM plus interferon inhalation	Protease inhibitors with cytokine as aerosol	50 patients suffering from pneumonia caused by COVID-19	NCT04291729	4
Xiyanping or lopinavir-ritonavir-interferon inhalation	Anti-inflammatory (Xiyanping) or Protease inhibitors with cytokine	384 patients with pneumonia caused by COVID-19	NCT04275388	N/A
Xiyanping combined with lopinavir + ritonavir	Anti-inflammatory (Xiyanping) in combination with Protease inhibitors	80 patients infected with COVID-19	NCT04295551	N/A
Combinations of oseltamivir, favipiravir, and chloroquine	Neuraminidase (Oseltamivir) in combination with antimalarial/ amebicide	80 patients infected with COVID-19	NCT04303299	3
Thalidomide	Angiogenesis inhibitor and immunomodulator	40 patients infected with COVID-19	NCT04273581	2
Thalidomide	Angiogenesis inhibitor and immunomodulator	100 patients suffering from pneumonia caused by COVID-19	NCT04273529	2
Vitamin C	Vitamin (Ascorbic acis)	140 patients with severe pneumonia caused by COVID-19	NCT04264533	2
Methylprednisolone	Corticosteroid	80 patients infected with COVID-19	NCT04244591	2
Pirfenidone	Pyridone	294 patients with severe pneumonia caused by COVID-19	NCT04282902	3
Bromhexine hydrochloride	Mucolytics	60 patients with suspected and mild pneumonia caused by COVID-19	NCT04273763	N/A
Bevacizumab	Monoclonal antibody	20 patients with COVID-19 associated with severe pneumonia	NCT04275414	2 and 3
Fingolimod	Sphingosine 1-phosphate receptor modulator	30 patients infected with COVID-19	NCT04280588	N/A

* Information source: clinicaltrial.gov. N/A = Not available.
